# Predicting Atrial Fibrillation Recurrence by Combining Population Data and Virtual Cohorts of Patient-Specific Left Atrial Models

**DOI:** 10.1161/CIRCEP.121.010253

**Published:** 2022-01-28

**Authors:** Caroline H. Roney, Iain Sim, Jin Yu, Marianne Beach, Arihant Mehta, Jose Alonso Solis-Lemus, Irum Kotadia, John Whitaker, Cesare Corrado, Orod Razeghi, Edward Vigmond, Sanjiv M. Narayan, Mark O’Neill, Steven E. Williams, Steven A. Niederer

**Affiliations:** School of Biomedical Engineering and Imaging Sciences, King’s College London, United Kingdom (C.H.R., I.S., J.Y., M.B., A.M., J.A.S.-L., I.K., J.W., C.C., O.R., M.O., S.E.W., S.A.N.).; School of Engineering and Materials Science, Queen Mary University of London, United Kingdom (C.H.R.).; The Department of Internal Medicine, Cardiovascular Division, Brigham and Women’s Hospital, Boston, MA (J.W.).; IHU Liryc, Electrophysiology and Heart Modeling Institute, Fondation Bordeaux Université, France (E.V.).; Univ. Bordeaux, IMB, UMR 5251, F-33400 Talence, France (E.V.).; Department of Medicine and Cardiovascular Institute, Stanford University, Palo Alto, CA (S.M.N.).; Centre for Cardiovascular Science, College of Medicine and Veterinary Medicine, University of Edinburgh (S.E.W.).

**Keywords:** atrial fibrillation, benchmarking, exercise test, machine learning, uncertainty

## Abstract

Current ablation therapy for atrial fibrillation is suboptimal, and long-term response is challenging to predict. Clinical trials identify bedside properties that provide only modest prediction of long-term response in populations, while patient-specific models in small cohorts primarily explain acute response to ablation. We aimed to predict long-term atrial fibrillation recurrence after ablation in large cohorts, by using machine learning to complement biophysical simulations by encoding more interindividual variability.

**Methods:**

Patient-specific models were constructed for 100 atrial fibrillation patients (43 paroxysmal, 41 persistent, and 16 long-standing persistent), undergoing first ablation. Patients were followed for 1 year using ambulatory ECG monitoring. Each patient-specific biophysical model combined differing fibrosis patterns, fiber orientation maps, electrical properties, and ablation patterns to capture uncertainty in atrial properties and to test the ability of the tissue to sustain fibrillation. These simulation stress tests of different model variants were postprocessed to calculate atrial fibrillation simulation metrics. Machine learning classifiers were trained to predict atrial fibrillation recurrence using features from the patient history, imaging, and atrial fibrillation simulation metrics.

**Results:**

We performed 1100 atrial fibrillation ablation simulations across 100 patient-specific models. Models based on simulation stress tests alone showed a maximum accuracy of 0.63 for predicting long-term fibrillation recurrence. Classifiers trained to history, imaging, and simulation stress tests (average 10-fold cross-validation area under the curve, 0.85±0.09; recall, 0.80±0.13; precision, 0.74±0.13) outperformed those trained to history and imaging (area under the curve, 0.66±0.17) or history alone (area under the curve, 0.61±0.14).

**Conclusion:**

A novel computational pipeline accurately predicted long-term atrial fibrillation recurrence in individual patients by combining outcome data with patient-specific acute simulation response. This technique could help to personalize selection for atrial fibrillation ablation.

## Abstract 2

Supplemental Digital Content is available in the text.

**What Is Known?**

- Current ablation therapy for atrial fibrillation is suboptimal, and long-term response is challenging to predict.
- Clinical trials identify bedside properties that provide only modest prediction of long-term response in populations, while patient-specific models in small cohorts primarily explain acute response to ablation.
- Identifying optimal ablation approaches for individual patients has the potential to improve safety, inform patient selection, and decrease time and cost for procedures.

**What the Study Adds**

- A novel computational pipeline to predict long-term atrial fibrillation recurrence in individual patients by combining outcome data with patient-specific acute simulation response.
- Including biophysical simulations metrics improved classifier performance; classifiers trained to history, imaging, and simulation metrics outperformed those trained to history and imaging or history alone.
- Our technique could help to personalize selection for atrial fibrillation ablation.

Radiofrequency catheter ablation therapy is recommended in symptomatic drug refractory atrial fibrillation patients. Atrial fibrillation ablation therapy ranges from pulmonary vein isolation to more extensive ablation strategies consisting of pulmonary vein isolation together with multiple additional lesions. Atrial fibrillation patients represent a diverse population requiring a range of different treatment approaches; no single approach is right for all patients, with suboptimal success from pulmonary vein isolation of 55% to 75% at 1.5 years.^1^ Identifying a priori optimal ablation approaches for individual patients has the potential to improve safety, inform patient selection, and decrease time and cost for procedures.

Large clinical trials evaluate standard ablation strategies to provide evidence on long-term treatment efficacy for the average patient in a cohort and to derive risk scores for estimating a patient’s risk of atrial fibrillation recurrence.^2–5^ However, such trials have provided only modest prediction using demographic information, imaging metrics,^4^ acute atrial fibrillation termination,^6^ or in multivariate regression analysis. Moreover, it is not clear how to apply these population data to an individual patient. As an emerging approach, patient-specific biophysical modeling studies enable simulation and comparison of multiple ablation approaches in a single patient^7–9^ but have largely been applied to small cohorts of relatively homogeneous patients,^10^ and it is unclear how to generalize such models for general clinical use.

We developed a novel computational approach to predict long-term response after ablation in large cohorts, by using machine learning to combine patient-specific models of atrial fibrillation, derived metrics of atrial fibrillation physiology, clinical demographics, and imaging data. We captured unknowns in patient properties such as type of fibrotic remodeling, fiber field, and electrical properties by performing a series of simulation model variant stress tests to evaluate the susceptibility of the atrial substrate to sustained atrial fibrillation. In this work, we aimed to (1) generate comprehensive patient-specific atrial fibrillation signatures from multiple biophysical simulation model variant stress tests for a cohort of 100 patients and (2) train a machine learning classifier to predict long-term ablation outcome from this patient-specific signature.

## Methods

The Methods are briefly described here with full details in the Supplemental Material. We have irreversibly anonymized the 100 models and made these available at https://cemrg.com/models.html.

### Patient Cohort

Cardiac magnetic resonance imaging (MRI) data were processed for 43 paroxysmal atrial fibrillation, 41 persistent atrial fibrillation, and 16 long-standing persistent atrial fibrillation patients undergoing imaging at St Thomas’ Hospital to create a total of 100 patient-specific models. Ethical approval was granted by the regional ethics committee (17/LO/0150 and 15/LO/1803), and subjects gave informed consent. The inclusion criteria for this study were first-time atrial fibrillation ablation patients with no previous left atrial ablation who had late-gadolinium enhancement MRI performed at the clinician’s discretion for preprocedural planning. At St Thomas’ Hospital, ablation treatment is indicated for patients with symptoms of atrial fibrillation who have failed a single antiarrhythmic agent. These patients underwent first-time catheter ablation therapy for atrial fibrillation, which consisted of pulmonary vein isolation alone or with the addition of ablation lines (mitral or roof) or posterior box isolation ablation.^11^ Patients were followed up for 1 year after their ablation procedure as per routine assessment at our institution. This consisted of 2 to 4 appointments over the year with AF symptom assessment, 12-lead ECG recordings, and ambulatory monitoring on the basis of patient symptoms. Atrial fibrillation recurrence was assessed following a 3-month blanking period. The Table details patient demographics, ablation therapy approach, and antiarrhythmic drug therapy, analyzed by atrial fibrillation recurrence.

**Table.**
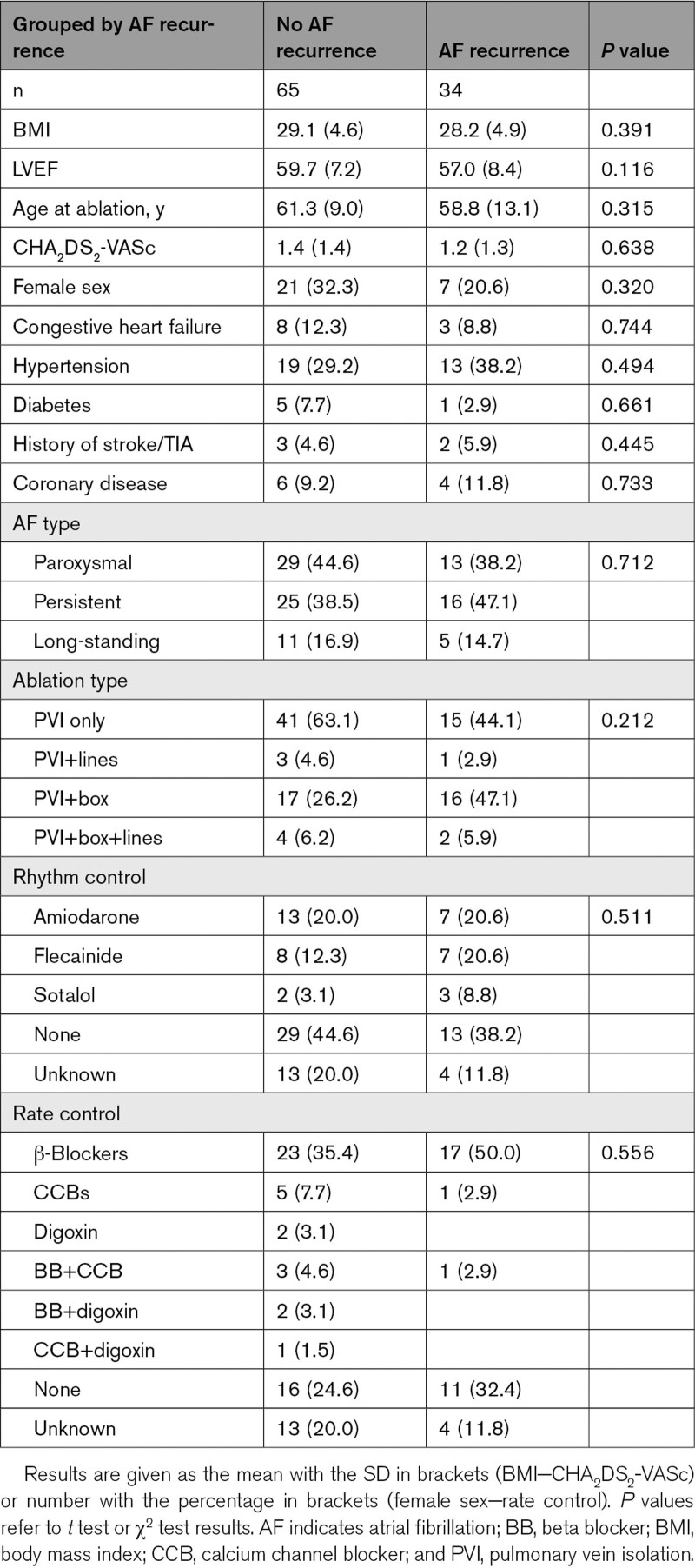
Clinical Metrics Analyzed by AF Recurrence

**Figure 1.**
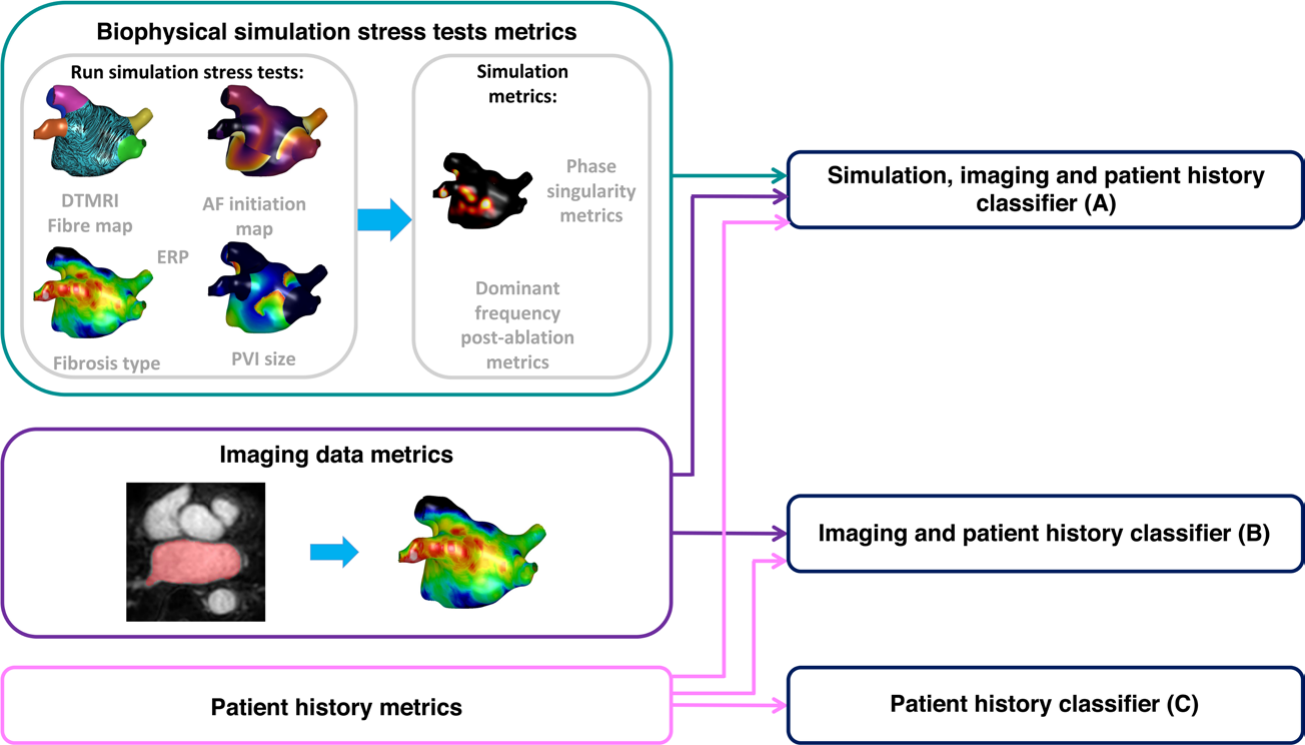
**Schematic methodology for using machine learning to combine biophysical simulation stress tests for acute simulation responses with population data to predict long-term atrial fibrillation (AF) recurrence.** Clinical imaging data were used to construct a cohort of patient-specific models. Biophysical simulation stress tests with different types of fibrosis, fiber maps, AF induction protocols, effective refractory period (ERP) values, and pulmonary vein isolation (PVI) sizes were used to test AF inducibility. These simulation stress test metrics were combined with imaging and patient history metrics to produce a patient-specific signature. This was repeated to produce a population of models. Machine learning classifiers were trained across this population to predict clinical outcome from patient-specific signature. Classifiers used either (**A**) simulation, imaging, and patient history metrics; (**B**) imaging and patient history metrics; or (**C**) patient history metrics. DT-MRI indicates diffusion tensor magnetic resonance imaging.

The schematic in Figure 1 shows an overview of the methodology used for predicting clinical outcome by combining patient-specific biophysical simulation stress tests and population data through machine learning techniques.

### Simulated Atrial Fibrillation Model Variant Stress Tests

**Figure 2.**
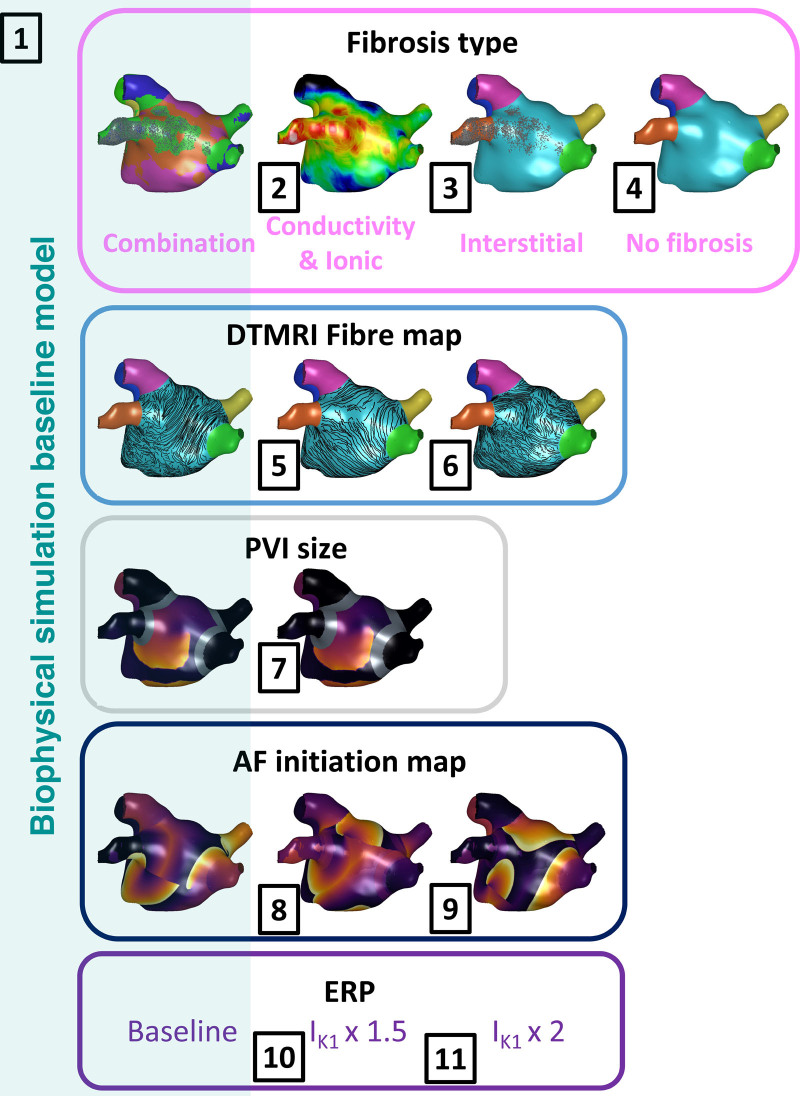
**Simulation model variant stress tests.** The choices indicated by the light blue background represent the baseline model. Other setups include the baseline model setup with a variation in one of the following model features: (setups: 2–4) fibrosis type, (5 and 6) diffusion tensor magnetic resonance imaging (DT-MRI) fiber maps, (7) pulmonary vein isolation size (PVI), (8 and 9) atrial fibrillation (AF) initiation map, (10 and 11) effective refractory period (ERP) values.

Models were constructed using the steps given in the Supplemental Material. Simulation model variant stress tests were designed to probe the uncertainty in atrial properties and test the ability of the substrate to sustain atrial fibrillation before and after varying ablation lesion sets. Acute response to simulated ablation was tested for 11 different simulation setups shown in Figure 2. The baseline setup, shown in the light blue box and numbered (1) in Figure 2, included combination fibrotic remodeling (interstitial fibrosis with conductivity and ionic changes) together with the baseline choice for the following properties: diffusion tensor MRI fiber field, pulmonary vein isolation lesion set, atrial fibrillation initiation map, and effective refractory period. To evaluate the effects of uncertainty in each component of the atrial substrate separately, we varied the properties of the baseline atrial model individually, while leaving the other properties of the model fixed at the baseline values. The properties we varied were the type of fibrotic remodeling (tests 1–4), the diffusion tensor MRI fiber map (5 and 6), the ablation lesion size (7), the initiation protocol (8 and 9), and the electrical properties (10 and 11). Preablation arrhythmia simulations (15 s) were analyzed for setups 1 to 4; postablation arrhythmia simulations (2 s) were analyzed for setups 1 to 11. More details on each setup are given in the Supplemental Material.

### Machine Learning Classifiers to Predict Atrial Fibrillation Recurrence on Long-Term Follow-Up

Machine learning classifiers were trained to map clinical data to long-term outcome. Specifically, classifiers were trained to predict binary clinical atrial fibrillation recurrence for 3 clinical data sets: (1) simulation, imaging, and patient history; (2) imaging and patient history; and (3) patient history alone. Further details on the metrics included in each classifier are given in the Supplemental Methods.

### Statistical Analysis

For each data set (1–3), the following machine learning classifiers were compared: K nearest neighbors, support vector machine, random forest, and logistic regression. Each classifier was trained to each data set either with or without principal component analysis preprocessing, with the number of components chosen to retain 95% of the variance. The accuracy, recall, precision, and receiver operating characteristic area under the curve values were compared for each combination of data set and classifier, with and without principal component analysis. For each data set (1–3), the classifier with the largest receiver operating characteristic area under the curve value was selected. Further details are given in the Supplemental Methods.

## Results

### Cohort Properties

Follow-up data were available for 99 of the 100 cases. AF recurred in the first year after ablation (following a 3-month blanking period) for 34 of the patients, with a mean recurrence time of 189±95 days. None of the clinical metrics considered were significantly different between cases with or without arrhythmia recurrence (Table).

### Imaging Metrics Related to Atrial Fibrillation Recurrence

**Figure 3.**
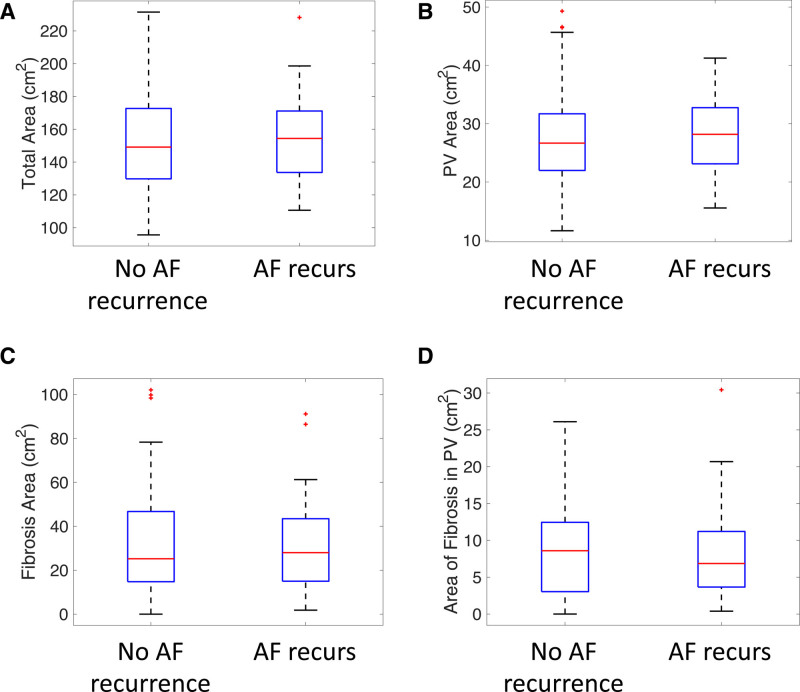
**Simple imaging metrics do not vary with atrial fibrillation (AF) recurrence.** **A**, Total surface area (*P*=0.55). **B**, Pulmonary vein (PV) surface area (*P*=0.58). **C**, Total fibrosis surface area (thresholded at image intensity ratio >1.22; *P*=0.94). **D**, Total fibrosis surface area in the PV regions (*P*=0.72).

The average visual fibrosis score was higher for the atrial fibrillation recurrence group, but this did not reach significance (*P*=0.169). Figure 3 shows that, when defining fibrotic regions with an image intensity ratio threshold of 1.22, the calculated imaging metrics were not significantly different between the groups with and without atrial fibrillation recurrence. These include (1) total atrial surface area (152.0±30.5 versus 154.8±27.2 cm^2^; *P*=0.55), (2) pulmonary vein surface area (27.8±8.6 versus 28.2±7.0 cm^2^; *P*=0.58), (3) fibrosis surface area (32.4±24.2 versus 31.9±22.0 cm^2^; *P*=0.94), and (4) area of fibrosis in the pulmonary veins (8.6±6.3 versus 6.3±6.6 cm^2^; *P*=0.72). The median fibrosis surface areas by visual fibrosis category were as follows: healthy, 23.7 cm^2^; mild, 18.2 cm^2^; moderate, 33.1 cm^2^; and severe, 41.6 cm^2^.

### Relating Acute Atrial Fibrillation Termination by Simulated Ablation to Long-Term Recurrence

**Figure 4.**
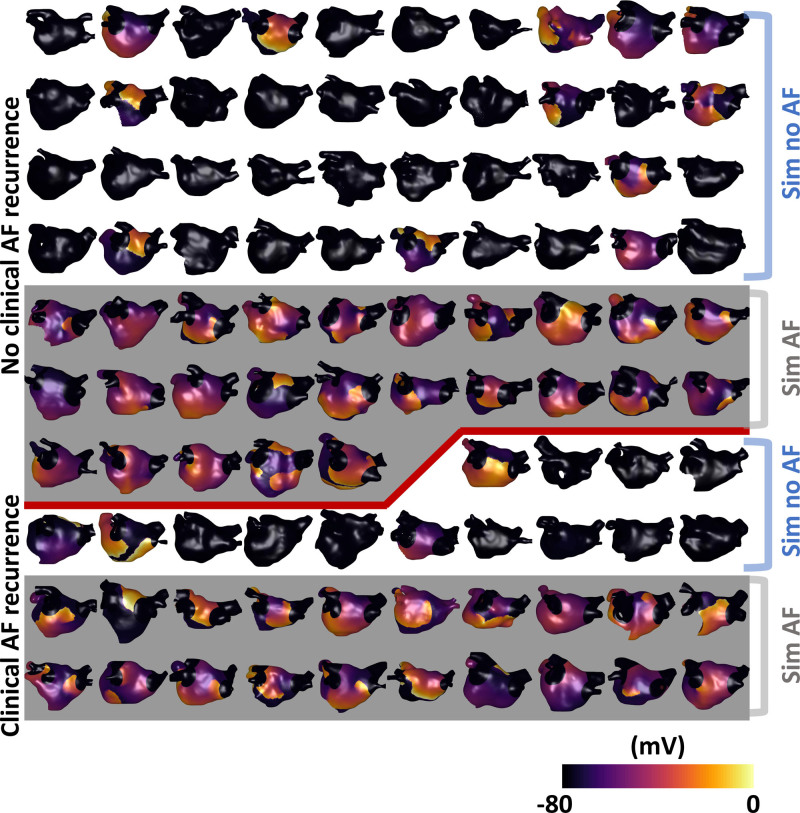
**Acute response to pulmonary vein isolation ablation for simulations incorporating interstitial fibrosis grouped by clinical atrial fibrillation (AF) recurrence.** Transmembrane potential plots are shown 2 s after pulmonary vein isolation ablation for the interstitial fibrosis simulation setup. The first 65 cases had no clinical AF recurrence, while the bottom 34 had AF recurrence. The background color indicates whether acute simulation response was considered successful (termination to sinus rhythm or organized nonfibrillatory rhythms) in white or AF is sustained in gray.

Prediction accuracy of the single acute simulation stress tests for predicting long-term clinical atrial fibrillation recurrence was in the range: 0.38 to 0.63 (using a threshold dominant frequency of 4.7 Hz to define simulations with atrial fibrillation). Figure 4 shows transmembrane potential maps 2 s after pulmonary vein isolation ablation for the interstitial fibrosis setup (simulation stress test setup number 3): 40 of 65 cases of no clinical recurrence were classified correctly, and 20 of 34 cases of clinical recurrence were classified correctly using the acute simulation outcome.

In general, acute simulation outcome stress tests did not differentiate between clinical outcomes. Table S1 gives all the simulation metrics by group (without or with clinical atrial fibrillation recurrence). The table first lists properties of the 15-s atrial fibrillation simulations before pulmonary vein isolation was applied for the different fibrosis type setups 1 to 4, as follows: mean number of phase singularities, phase singularity area, and pulmonary vein phase singularity area. These are followed by the outcome variables given as dominant frequency (atrial rate) for the simulations in the 2 s after pulmonary vein isolation was applied for setups 1 to 11. For dominant frequency, there was a trend between groups without and with atrial fibrillation recurrence for simulations including interstitial fibrosis (setup number 3: 2.6±2.5 versus 3.4±2.3 Hz; *P*=0.11) and no fibrotic remodeling (setup number 4: 3.2±2.5 versus 4.1±2.1 Hz; *P*=0.095). Other simulation metrics were not significantly different.

### Prediction of Atrial Fibrillation Recurrence by Combining Population Data and Patient-Specific Modeling

**Figure 5.**
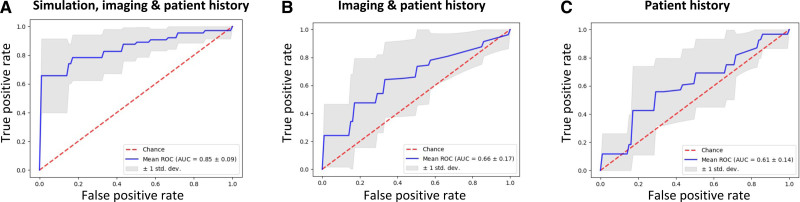
**Receiver operating characteristic (ROC) curves for simulation, imaging, and patient history classifiers.** ROC curves for classifiers constructed from (**A**) simulation, imaging, and patient history data (support vector machine classifier); (**B**) imaging and patient history data (K nearest neighbor classifier); and (**C**) patient history data alone (random forest classifier). The gray area indicates ±1 SD calculated from 10-fold cross-validation. AUC indicates area under the curve.

Figure 5 shows receiver operating characteristic curves for optimal classifiers constructed from (1) simulation, imaging, and patient history data; (2) imaging and patient history data; and (3) patient history data.

For the simulation, imaging, and patient history classifier (Figure 5A), the optimal classifier was support vector machine with principal component analysis: receiver operating characteristic (ROC) area under the curve (AUC), 0.85±0.09; accuracy, 0.74±0.13; recall, 0.80±0.12; and precision, 0.72±0.15. Other classifier ROC AUC values were as follows: K nearest neighbor, 0.85±0.09; random forest, 0.77±0.14; and logistic regression, 0.59±0.12.

Conversely, less inclusive classifiers were less predictive. Figure 5B shows results for the imaging and patient history classifier; the optimal classifier in this case was K nearest neighbor with principal component analysis: ROC AUC, 0.66±0.17; accuracy, 0.68±0.07; recall, 0.57±0.34; and precision, 0.58±0.38. For the patient history classifier shown in Figure 5C, the random forest classifier was optimal: ROC AUC, 0.61±0.14; accuracy, 0.64±0.14; recall, 0.46±0.24; and precision, 0.46±0.28.

## Discussion

### Main Findings

We present a novel personalized digital approach that predicted response to atrial fibrillation ablation in individual patients when patient-specific geometry and simulations were combined with clinical data. The foundation for this approach demonstrates a novel computational pipeline that can be tuned to individual patient features, which takes into account likely physiological interactions between clinical demographics and the natural history of atrial fibrillation post-ablation and which can be readily scaled to personalize therapy. Notably, we found that predicting atrial fibrillation ablation response was suboptimal based on patient history or imaging data alone. Adding patient-specific simulations significantly improved prediction accuracy. This is the largest atrial fibrillation simulation study to date, demonstrating that patient-specific simulation can be scaled to generate virtual cohorts that can predict patient-level outcomes and could potentially be used to design optimal procedures for each individual a priori.

### Comparison With Other Imaging Predictors of Atrial Fibrillation Recurrence

Translating from average results to predictions for individual patients using standard risk scores is challenging. Previous studies have assessed the utility of anatomic and imaging metrics calculated from populations of images for predicting atrial fibrillation recurrence. For example, the DECAAF clinical trial (Delayed-Enhancement MRI Determinant of Successful Radiofrequency Catheter Ablation of Atrial Fibrillation) indicated that the degree of atrial late-gadolinium enhancement was independently associated with atrial fibrillation recurrence following catheter ablation in a cohort of 260 patients.^12^ We did not find this in our study; however, we used a smaller cohort with both paroxysmal and persistent atrial fibrillation patients. For anatomic metric analysis, Varela et al^2^ analyzed left atrial anatomy from MRI across a cohort of 144 patients to predict atrial fibrillation recurrence using vertical asymmetry together with left atrial sphericity to give an area under the ROC curve of 0.71. Bratt et al^3^ demonstrated that atrial volume is a good predictor of atrial fibrillation recurrence, with an ROC AUC of 0.77. They automatically segmented the left atrial body from computed tomography scans using deep learning and showed that atrial volume is an independent predictor of atrial fibrillation, with an age-adjusted relative risk of 2.9.^3^ Costa et al^4^ showed that left atrial volume is more important than atrial fibrillation type for predicting atrial fibrillation recurrence following pulmonary vein isolation. In contrast to these studies, Ebersberger et al^13^ showed no association between pulmonary vein properties or left atrial anatomic or functional properties measured on computed tomography and early atrial fibrillation recurrence at 3 to 4 months post-ablation. Our study also found that simple imaging metrics are not predictive of atrial fibrillation recurrence. However, we did not include vertical asymmetry or volume in this assessment, and we used MRI rather than computed tomography data.^14^

Computed tomography data also provide information on epicardial adipose tissue content, which may affect atrial fibrillation maintenance. Nalliah et al^15^ investigated the mechanisms for how epicardial adipose tissue affects atrial fibrillation, showing that higher adipose tissue is associated with slower conduction, higher degrees of electrogram fractionation, increased fibrosis, and increased lateralization of connexin40 gap junctional protein. Further to this, El Mahdiui et al^5^ found that posterior left atrial adipose tissue attenuation is predictive of atrial fibrillation recurrence post-ablation.

### Comparison With Other Simulation Predictors of Atrial Fibrillation Recurrence

Shade et al^10^ combined modeling and machine learning to predict atrial fibrillation recurrence in a cohort of 32 patients with paroxysmal atrial fibrillation. This study extends their elegant work by testing a range of unknowns in the substrate, enabling a greater degree of personalization through a simulation stress test approach, and by testing the effects of ablation approach, in a larger cohort of less homogeneous paroxysmal and persistent atrial fibrillation patients. The simulation stress test approach used in our study is analogous to a rigorous clinical test of postpulmonary vein isolation atrial fibrillation inducibility, which provided high specificity for atrial fibrillation recurrence in a large meta-analysis^16^ although it is difficult to apply due to practical constraints. We used a technique of initiating reentry through seeding phase singularities in multiple different locations. We applied this technique to initiate atrial fibrillation in setups 1 to 4 before ablation and also to test inducibility after pulmonary vein isolation for setups 1 to 11. This technique is more computationally efficient but may be less clinically realistic than the initiation technique of rapid pacing from multiple locations performed by Boyle et al.^9^ Recently, Azzolin et al^17^ proposed a technique that paces at the end of the effective refractory period to initiate atrial fibrillation and compared this to rapid pacing or using a phase distribution method to show that their method induced a larger variety of reentry scenarios, with a marginal increase in simulation time. More extensive inducibility testing protocols, such as those proposed by Boyle et al^9^ and Azzolin et al,^17^ could be used to identify further reentry areas and as additional features for the classifiers, which may increase the predictive accuracy.

### Limitations

There are multiple factors we did not include in the simulation model including the effects of ectopic beats on arrhythmia recurrence. We did not model the pulmonary vein isolation ablation lesions applied clinically but rather simulated these lesions as wide area circumferential ablation at a fixed distance from the left atrial/pulmonary vein junctions. Further, these lesion sets may be incomplete with gaps of surviving or recovered tissue, which would affect acute simulation outcome. We only simulated pulmonary vein isolation and did not include patient-specific lesion sets. We considered follow-up data for 1 year post-ablation only. The choice of image intensity threshold used for modeling scar will influence the imaging and simulation metrics. We used rule-based calibration of conduction velocity based on image intensities, but there is uncertainty associated with this prediction. We do not have validation of this rule-based inclusion of patient-specific electrophysiology across the data set used in the current study.^18^ We only included the left atrium in our simulations; however, performing biatrial simulations^19–21^ may improve the predictive accuracy of the classifier. Adding features derived from the 12-lead ECG provides additional information on the atria and could further improve the classifier.^7^ Overall, further work is required to choose the optimal simulation stress test setup. The optimal classifier properties for screening for likely atrial fibrillation recurrence will be considered in future studies.

### Conclusions

We present a novel computational pipeline that accurately predicted atrial fibrillation recurrence following ablation therapy in individual patients by combining outcome data with patient-specific acute simulation response. This technique could help to personalize selection for atrial fibrillation ablation and could be evaluated through a prospective clinical trial.

## Article Information

### Sources of Funding

Dr Roney acknowledges a Medical Research Council Skills Development Fellowship (MR/S015086/1). Dr Niederer acknowledges support from the UK Engineering and Physical Sciences Research Council (EP/M012492/1, NS/A000049/1, and EP/P01268X/1), the British Heart Foundation (PG/15/91/31812 and PG/13/37/30280), and Kings Health Partners London National Institute for Health Research (NIHR) Biomedical Research Centre. This work was supported by the Wellcome/EPSRC Centre for Medical Engineering (WT 203148/Z/16/Z). This study received financial support from the French Government as part of the “Investments of the Future” program managed by the National Research Agency (ANR), grant reference ANR-10-IAHU-04. Dr Narayan acknowledges support from NIH R01 HL149134 (Machine Learning in Atrial Fibrillation) and R01 HL83359 (Dynamics of Atrial Fibrillation). Dr Williams acknowledges support from a British Heart Foundation Intermediate Clinical Research Fellowship (FS/20/26/34952).

### Disclosures

M. O’Neill has received research support and honoraria from Biosense Webster and has received consultation fees from Medtronic, Biosense Webster, St. Jude/Abbott, and Siemens. Dr Williams has received research support from Biosense Webster and EPD Solutions and consulting fees from Imricor Medical Systems. Dr Niederer reports intellectual property rights from King’s College London and support from Siemens, Pfizer, EBR Systems, Boston Scientific, and Abbott. Dr Narayan reports consulting from Beyond.ai, Inc, TDK, Inc, Up to Date, Abbott Laboratories, and the American College of Cardiology Foundation and intellectual property rights from the University of California Regents and Stanford University. The other authors report no conflicts.

### Supplemental Material

Supplemental Methods

Table S1

Videos S1 and S2

References ^22–48^

## Supplementary Material


