# Supplementary material for: Predicting Atrial Fibrillation Recurrence by Combining Population Data and Virtual Cohorts of Patient-Specific Left Atrial Models
Source: Circ Arrhythm Electrophysiol. 2022 Jan 28;15(2):e010253. doi: 10.1161/CIRCEP.121.010253 (PMC8845531; doi:10.1161/CIRCEP.121.010253)
Supplement: Supplementary file 1 [file hae-15-e010253-s001.pdf]

SUPPLEMENTAL MATERIAL

Supplemental Methods

Construction of patient specific models

For each case, the model anatomy and fibrosis were personalised to the patient's MRI data to give a patient-specific model. To achieve this, the left atrium was segmented from a contrast enhanced magnetic resonance angiogram scan and then registered with the corresponding late-gadolinium enhancement magnetic resonance imaging (LGE-MRI) scan using our previously reported CEMRGApp [22]. The resulting mesh was post-processed using a sequence of steps to create a mesh suitable for simulation studies. First, the following sequence of filters were applied using Meshlab software to create a closed surface: Poisson surface reconstruction, marching cubes, and quadric edge collapse decimation [23]. The closed surface mesh was then clipped at the mitral valve and four pulmonary veins using Paraview software (Kitware, Clifton Park, NY, USA [24]), and remeshed using mmgtools software (www.mmgtools.org [25,26]) to a regular average edge length of 0.34mm suitable for electrophysiology simulations. Meshes were labelled to include each of the four pulmonary veins and the left atrial appendage [27]. Finally, this endocardial surface mesh was duplicated and projected an arbitrarily small distance (0.1mm) epicardially to construct a separate epicardial mesh for a bilayer model. The two surfaces were joined with linear elements. A homogeneous conductivity is assigned to these transmural elements that reflects the wall thickness following Labarthe et al. [28].

Simulations used the Courtemanche et al human atrial cell model with atrial fibrillation electrical remodelling [29] and the monodomain solver within the CARPentry cardiac electrophysiology simulation software [30]. Repolarisation heterogeneity was included in the

models as different maximal ionic conductances in the pulmonary vein and appendage regions [31]; parameter values are given in Bayer et al [32].

Atrial fibres from a fibre atlas were registered to each anatomy using the universal atrial coordinate system [27]. The fibre atlas used corresponded to the endocardial and epicardial surface fibres of a human ex-vivo DT-MRI dataset [33]. This fibre field was chosen because it was the most representative of the fibre fields tested (the individual fibre fields in the DT-MRI dataset and the average fibre field) for left atrial arrhythmia simulations.

Fibrotic remodelling methodology

The effects of fibrotic remodelling were included for each anatomy according to LGE-MRI intensity values. Fibrotic remodelling was modelled as regions of conduction slowing (structural remodelling) together with electrophysiological changes (electrophysiological remodelling) as follows. For structural remodelling, tissue conductivities were calibrated according to LGE-MRI image intensity ratio based on previously-determined values [34]. The conductivity values were as follows: IIR<0.9: 0.4S/m (CV: 0.81m/s), 0.9<IIR<1.1: 0.31S/m (CV: 0.74m/s), 1.1<IIR<1.4: 0.31S/m (CV: 0.74m/s), 1.4<IIR<1.6: 0.28S/m (CV: 0.71m/s), 1.6 < IIR: 0.19S/m (CV: 0.58m/s). Tissue was binarised depending on image intensity ratio into healthy (image intensity ratio <1.22) or fibrotic regions (image intensity ratio >1.22) [9]. For electrophysiological remodelling, ionic properties were modified in fibrotic regions to represent the effects of elevated TGF-ß1 (maximal ionic conductances were rescaled as follows: 50% g_{K1}, 60% g_{Na} and 50% g_{CaL}) [35,36].

Interstitial fibrosis was modelled using a probabilistic approach using the image intensity ratio from the LGE-MRI as an input. Mesh edges were randomly assigned to be fibrotic with

probability depending on the image intensity ratio value and the angle of the edge compared with the element fibre direction (edges in the longitudinal fibre direction were four times more likely to be selected than those in the transverse direction) [35]. Following our previous study, this was implemented using the scaling factor $\alpha(4\cos^2(\theta) + \sin^2(\theta))$, where θ is the angle between the mesh element edge and longitudinal fibre direction, and α was chosen to give a median length of connected interstitial fibrosis edges of 670 μm in regions with image intensity ratio > 1.22 [32,37]. Each edge of the mesh was assigned a uniformly distributed random number in the interval (0, 1) and this was compared to the product of the image intensity ratio and the previously defined scaling factor. If this product was greater than the random number, the edge was assigned to be fibrotic. No flux boundary conditions were applied along fibrotic edges to model microstructural discontinuities [38]. Combination fibrotic remodelling included interstitial fibrosis together with conductivity and ionic changes.

Atrial fibrillation induction protocols

To initiate arrhythmia in each patient specific model using comparable parameters and to reduce computation time, simulations were initiated with four spiral wave re-entries [8,39]. These conditions were defined identically for the endocardial and epicardial surfaces using the universal atrial coordinate system [27] as an activation time field with two Archimedean spirals on each of the posterior and anterior walls, with opposite chirality for adjacent spirals.

Modelling of ablation lesion sets

Pulmonary vein isolation was modelled as wide area circumferential ablation, consisting of two non-conducting rings (tissue conductivity: 0.001 S/m) at a distance of 6mm around the left and right LA-PV antra. Pulmonary vein antra were defined by segmentation blinded to electrophysiological results. We tested the acute impact of pulmonary vein isolation ablation

on atrial fibrillation induced using the protocols described above by determining the atrial rate, quantified by the dominant frequency, 2 seconds after ablation.

Post-processing atrial fibrillation simulations and calculating structural and electrical metrics

For each pre-ablation arrhythmia simulation, phase singularities were calculated for 15 seconds of arrhythmia data, or until termination if this occurred within 15 seconds [40]. Spatial phase singularity density maps were then calculated using our previous methodology [35].

For each left atrial mesh, the following structural metrics were calculated: left atrial surface area and fibrosis surface area (area >1.22 image intensity ratio). For each atrial fibrillation simulation, the following electrical metrics were calculated: average number of phase singularities, phase singularity surface area (area >1 standard deviation above mean phase singularity density) and pulmonary vein phase singularity density ratio (the density of phase singularities in the pulmonary vein region compared to the whole left atrium).

Steps towards validation of the modelling pipeline

The different components and spatial scales of the simulation pipeline have been tested in previous studies as follows. The cell model we used was the Courtemanche et al. human atrial cell model, with parameters chosen following Krummen et al. to match their human action potential duration restitution recordings [41]. Wilhelms et al. performed a benchmarking study of different atrial cell models including the Courtemanche et al. model to check the mathematical properties and ability of the models to reproduce electrophysiological phenomena, such as action potential alternans [42]. The monodomain model of cardiac tissue used in this study is a simplification of the bidomain model. Multiple studies have

demonstrated similar propagation patterns and spiral wave trajectories between monodomain and bidomain simulations[43,44], motivating the use of the computationally simpler monodomain model for our study. CARPentry cardiac electrophysiology simulation code was verified by Niederer et al. in a benchmarking study of cardiac electrophysiology simulators [45].

We personalised atrial models to the patient's MRI data to give a patient-specific model. We have previously demonstrated that segmentation of the atrial anatomy and atrial fibrosis assessment from LGE-MRI data is reproducible [22]. We used a rule-based calibration of the conductivity values of the models to LGE-MRI image intensity ratio based on our previous study where we had paired electroanatomic mapping conduction velocity maps and LGE-MRI data for 10 AF patients [34]. We performed simulations with different fibre fields derived from human ex vivo DT-MRI fibre fields [46]. However, we do not have validation of this rule-based inclusion of patient-specific electrophysiology across the dataset used in the current study.

Simulated atrial fibrillation model variant stress tests

Simulation model variant stress tests were designed to probe the uncertainty in atrial properties and test the ability of the substrate to sustain atrial fibrillation before and after varying ablation lesion sets (see **Figure 2** of the main manuscript). Specifically, the effects of uncertainty in fibrotic remodelling were evaluated by simulating the following model variants: (1) combination remodelling (the baseline model, which includes interstitial fibrosis with conductivity and ionic changes); (2) conductivity and ionic remodelling only; (3) interstitial fibrosis only; (4) no fibrotic remodelling. For simulation set-up (5), the fibre field was changed to the average endocardial and epicardial DTMRI fibre fields [46], and for (6) the

fibre field was changed to the most different of the endocardial and epicardial DTMRI fibre fields (from field 1 to field 3 [46]). For simulation set-up (7), the effects of pulmonary vein isolation ablation area were tested by simulating a larger pulmonary vein isolation lesion set. For simulation set-ups (8) and (9), the effects of initial condition on acute outcomes were tested by modelling two additional atrial fibrillation induction protocols. Specifically, we used two additional arrangements of four spiral wave re-entries that were chosen since these revealed the greatest number of additional phase singularity sites in testing. For simulation set-ups (10) and (11), the effects of electrical properties and effective refractory period were investigated by multiplying I_{K1} conductance by 1.5 or 2, respectively.

Machine learning classifiers to predict atrial fibrillation recurrence on long-term follow up

Machine learning classifiers were trained to map clinical data to long-term outcome. Specifically, classifiers were trained to predict binary clinical atrial fibrillation recurrence for three clinical datasets: (a) simulation, imaging and patient history, (b) imaging and patient history, (c) patient history alone.

The simulation metrics used included number of phase singularities and pulmonary vein phase singularity density ratio for both the baseline simulation set-up (1: combination fibrosis) and averaged across the fibrotic remodelling set-ups (1-4); together with dominant frequency measured 2 seconds post-ablation for each of the eleven simulation set-ups (1-11).

The imaging metrics used were visual fibrosis score (assessed visually by two expert clinicians to give a score of either no fibrosis (scored as 0), mild fibrosis (1), moderate

fibrosis (2) or severe fibrosis (3)); total left atrial surface area; total pulmonary vein surface area; fibrosis area; and area of fibrosis in the pulmonary veins.

The patient history metrics considered were left ventricular ejection fraction, body mass index, age, female sex, congestive heart failure, history of hypertension, diabetes, history of stroke, coronary disease, and atrial fibrillation type (whether the patient had paroxysmal, persistent, or long-standing persistent atrial fibrillation). These patient demographics and history were documented on the clinical records.

Classifier (a) used the previously defined simulation metrics, together with the imaging metrics and the following patient history metrics: left ventricular ejection fraction, body mass index, age, CHA_2DS_2-VASc score and atrial fibrillation type. Classifier (b) used the imaging metrics and the same patient history metrics as classifier (a). Classifier (c) used an extended list of patient history metrics including the separate components of the CHA_2DS_2-VASc score; the metrics used were as follows: left ventricular ejection fraction, body mass index, age, CHA_2DS_2-VASc score, female gender, congestive heart failure, hypertension, diabetes, history of stroke, coronary disease and atrial fibrillation type. A subset of these patient history metrics was used for classifiers (a) and (b) to minimise the number of features and because these additional metrics are factored into the CHA_2DS_2-VASc score.

The additional benefit of including ablation type in the classifier predictions was assessed by adding the ablation type to each of the classifiers (0: pulmonary vein isolation, 1: pulmonary vein isolation plus lines, 2: pulmonary vein isolation plus posterior box isolation, 3: pulmonary vein isolation plus box plus lines).

Statistical Analysis

To select hyperparameters for each set-up, we performed 10-fold cross validation with a balanced-accuracy criterion. Each classifier was then trained for each dataset with the optimal hyperparameters, using 10-fold cross validation to calculate average accuracy, precision, recall and receiver operating characteristic curves. This was performed using scikit-learn in python, using the GridSearchCV and cross-validate packages [47]. TableOne was used to generate summary statistics tables [48]. Data are presented as mean ± standard deviation, and means are compared by the Student's t test or Chi-squared test (if categorical).

Supplemental Results

Prediction of atrial fibrillation recurrence by combining population data and patient-specific modelling: Effects of ablation type

Adding ablation type to the classifiers did not significantly change the metrics for the optimal classifiers. For the simulation, imaging and patient history classifier: the optimal classifier was support vector machine with principal component analysis: ROC AUC 0.84 ± 0.09, accuracy 0.73 ± 0.09, recall 0.81 ± 0.14, and precision 0.72 ± 0.14. For the imaging and patient history classifier, the optimal classifier in this case was K nearest neighbour with principal component analysis: ROC AUC 0.65 ± 0.13, accuracy 0.68 ± 0.11, recall 0.51 ± 0.26, and precision 0.58 ± 0.44. For the patient history classifier, the random forest classifier was optimal: ROC AUC 0.67 ± 0.16, accuracy 0.59 ± 0.07, recall 0.39 ± 0.22, and precision 0.39 ± 0.28.

Supplemental Discussion

Comparison with other simulation predictors of atrial fibrillation recurrence

The electrical changes applied in simulation set-ups 10 and 11 modified the mean cycle length from 202.4±14.1ms to 136.3±18.6ms and 116.2±18.6ms. This is a larger range than the action potential duration changes simulated by Deng et al. [18] who showed that such changes could change which fibrotic regions anchor re-entry. We see changes in phase singularity location with these electrical changes, and an increase in the number of phase singularities with shorter cycle lengths.

Mechanistic implications of our findings

For some cases, anatomical and imaging metrics were not informative for predicting atrial fibrillation recurrence, while acute simulation outcomes were more indicative. **Supplemental video I** shows simulation stress test acute simulations for a case with a low left atrial surface area and low fibrosis surface area for which atrial fibrillation recurred clinically, where ten of the eleven stress tests show atrial fibrillation. Conversely, **Supplemental video II** shows a case with a high left atrial surface area and high fibrosis surface area (which thus may be expected to have atrial fibrillation recurrence), for which atrial fibrillation did not recur clinically. For this case, only three of the eleven stress tests demonstrate atrial fibrillation recurrence. This shows the additional benefit of biophysical simulations for predicting atrial fibrillation recurrence.

Effects of ablation approach on atrial fibrillation recurrence

Our study used a retrospective dataset in which the ablation lesion set for each patient was chosen by the cardiac electrophysiologist. It is likely that the ablation lesion is informed by the patient demographics and imaging data. As such, adding the ablation lesion type to the

classifier may not provide any further information for improving predictions. Our data are not sufficient for making firm conclusions on this; to fully investigate the effects of ablation lesion set on atrial fibrillation recurrence would require data from a randomised clinical trial. With such data, the simulation stress tests could be extended to include patient-specific lesion sets, or to see if the classifier prediction changes with more extensive ablation lesion sets. Using the classifier together with a minimal path algorithm could enable fast prediction of estimated optimal lesion sets tailor ablation patterns to the individual patient.

Supplemental Table I

Grouped by AF recurrence	No AF recurrence	AF recurs	P-Value
n	65	34	
Mean PS 1	3.4 (1.1)	3.5 (1.6)	0.691
PS Area 1	11.9 (4.5)	11.4 (5.0)	0.638
PVPS Ratio 1	0.2 (0.2)	0.2 (0.2)	0.900
PS No 2	5.1 (2.3)	5.2 (2.6)	0.834
PS Area 2	14.2 (4.6)	14.0 (4.9)	0.857
PVPS Ratio 2	0.3 (0.1)	0.3 (0.1)	0.950
PS No 3	3.0 (1.4)	3.1 (1.2)	0.729
PS Area 3	11.1 (5.2)	10.9 (4.7)	0.849
PVPS Ratio 3	0.3 (0.2)	0.3 (0.2)	0.968
PS No 4	2.7 (0.9)	2.7 (1.0)	0.942
PS Area 4	13.8 (4.2)	13.1 (4.4)	0.504
PVPS Ratio 4	0.2 (0.1)	0.2 (0.1)	0.901
Mean PS No	3.6 (1.0)	3.6 (1.2)	0.812
Mean PVPS Ratio	0.2 (0.1)	0.2 (0.1)	0.838
DF1	3.1 (2.4)	3.2 (2.3)	0.808
DF2	2.3 (2.5)	2.8 (2.4)	0.403
DF3	2.6 (2.5)	3.4 (2.3)	0.114
DF4	3.2 (2.5)	4.1 (2.1)	0.095
DF5	2.5 (2.5)	3.0 (2.4)	0.291
DF5	1.9 (2.4)	2.7 (2.5)	0.142
DF7	2.3 (2.5)	2.7 (2.5)	0.479
DF8	4.1 (1.9)	3.7 (2.5)	0.525
DF9	2.1 (2.5)	1.8 (2.4)	0.520
DF10	6.8 (2.2)	6.6 (2.6)	0.710
DF11	7.9 (2.7)	7.7 (3.1)	0.699

Supplemental Table I: Simulation metrics calculated for cases without or with clinical AF recurrence 1-year post-ablation therapy.

PS No = Mean number of phase singularities; PS Area = Area of phase singularities; PVPS Ratio = PV phase singularity density ratio; DF = acute simulation mean dominant frequency 2 seconds post-ablation. The top of the table lists properties of the 15s atrial fibrillation simulations before pulmonary vein isolation was applied for the different fibrosis type set-ups 1-4, as follows: mean number of phase singularities, phase singularity area and pulmonary vein phase singularity area. Mean phase singularity number and mean pulmonary vein phase singularity ratio indicates the mean calculated across simulation set-ups 1-4. The bottom part of the table is the outcome variables given as dominant frequency (atrial rate) for the simulations in the 2 seconds after pulmonary vein isolation was applied. Numbers 1-11 refer to the simulation stress test set-ups in ***Figure 2****. Results are given as the mean with the standard deviation in brackets. P-values refer to t-test results.*

Supplemental video I: Simulation model variant stress tests for a case with clinical atrial fibrillation recurrence. *The different panels show simulation set-ups 1-11 from* ***Figure 2*** *together with the LGE-MRI intensity map. These videos are shown in the anteroposterior view for the 2s after pulmonary vein isolation is applied. Ten of the eleven cases show sustained arrhythmia.*

Supplemental video II: Simulation model variant stress tests for a case without clinical atrial fibrillation recurrence. *The different panels show simulation set-ups 1-11 from* ***Figure 2*** *together with the LGE-MRI intensity map. These videos are shown in the posteroanterior view for the 2s after pulmonary vein isolation is applied. Three of the eleven cases show sustained arrhythmia.*
