# Supplementary material for: Predicting Atrial Fibrillation Recurrence by Combining Population Data and Virtual Cohorts of Patient-Specific Left Atrial Models
Source: Circ Arrhythm Electrophysiol. 2022 Jan 28;15(2):e010253. doi: 10.1161/CIRCEP.121.010253 (PMC8845531; doi:10.1161/CIRCEP.121.010253)
Supplement: Supplementary file 4 [file hae-15-e010253-s004.pdf]

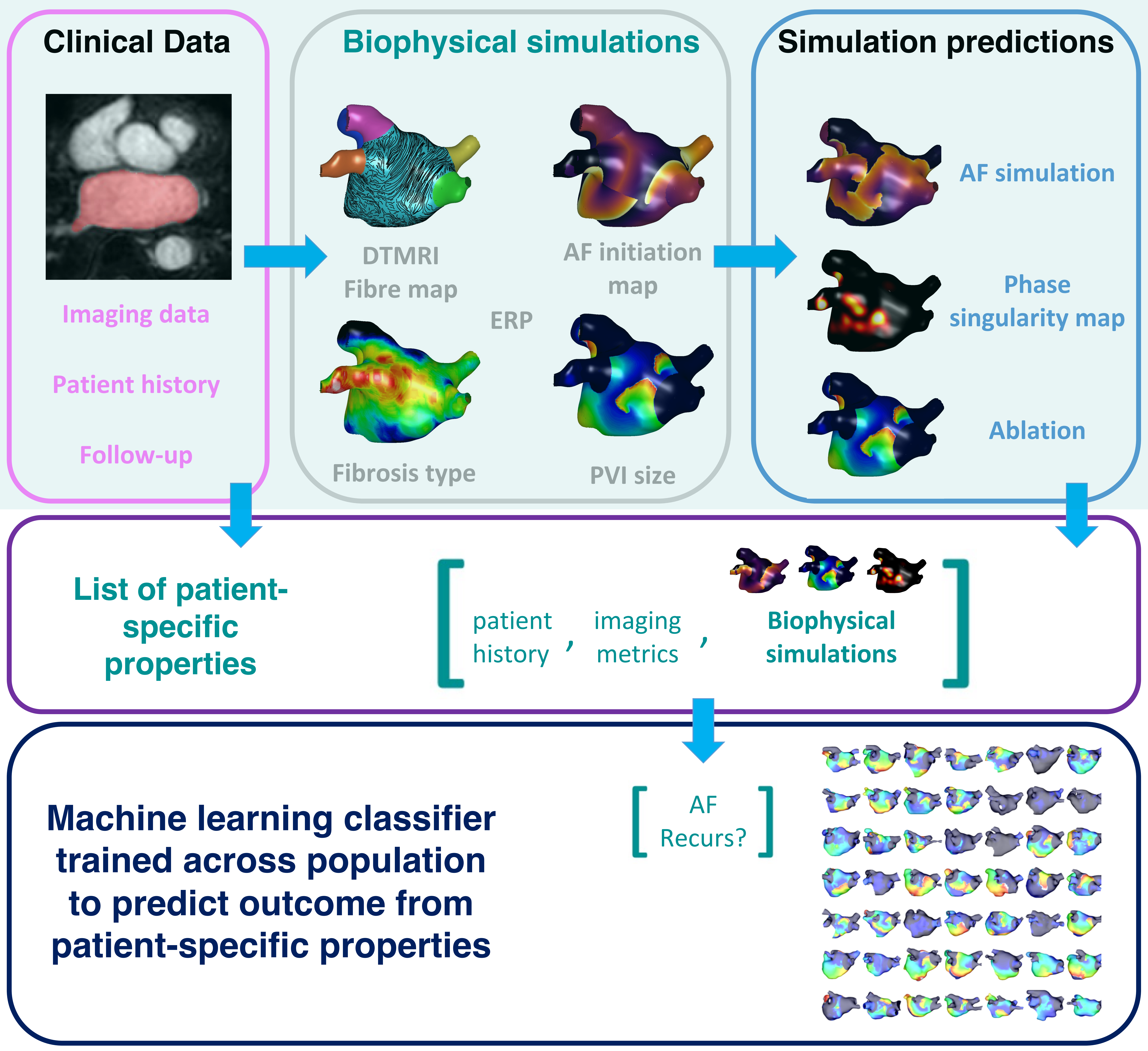
Clinical Data
Imaging data
Patient history
Follow-up
Biophysical simulations
DTMRI
Fibre map
AF initiation
map
ERP
Fibrosis type
PVI size
Simulation predictions
AF simulation
Phase
singularity map
Ablation
List of patient-
specific
properties
patient
history
,
imaging
metrics
,
Biophysical
simulations
Machine learning classifier
trained across population
to predict outcome from
patient-specific properties
AF
Recurs?
